# Delayed-onset cytomegalovirus retinitis after 0.2 µg/day fluocinolone acetonide implantation: a case series

**DOI:** 10.1186/s12348-026-00609-7

**Published:** 2026-06-24

**Authors:** Anamika Patel, Ilaria Testi, Carlos Pavesio

**Affiliations:** Uveitis Department, Moorfields Eye Hospital, 162 City Rd, London, EC1V 2PD UK

**Keywords:** CMV retinitis, ILUVIEN, Fluocinolone implant, Intravitreal steroids, Uveitis

## Abstract

**Purpose:**

To describe the clinical features and outcomes of cytomegalovirus retinitis (CMVR) presenting after intravitreal fluocinolone acetonide (FAc) implantation.

**Methods:**

Single centre, retrospective observational case series involving three patients who developed CMVR several months after FAc implantation at the uveitis department at Moorfields Eye Hospital, London, UK. Clinical history, immune status, ocular findings, imaging, and management were reviewed.

**Results:**

The first patient with pulmonary sarcoidosis and no systemic treatment presented 14 months post-implant with granular retinitis and severe occlusive retinal vasculitis. He developed renal toxicity to valganciclovir, necessitating intravitreal foscarnet and FAc implant removal. The second patient with presumed ocular sarcoidosis on multiple immunosuppressive agents developed fulminant necrotising CMVR eight months after FAc implantation and responded well to systemic valganciclovir with adjunctive intravitreal foscarnet therapy. The third patient, an elderly man with previous CMV retinitis in the context of follicular lymphoma, experienced reactivation at the margin of an old scar several months after FAc implantation and was stabilised with intravenous and intravitreal foscarnet. Although two patients had bilateral implants, the retinitis remained unilateral. Across cases, presentations ranged from granular retinitis with vasculitis to fulminant necrosis and late reactivation.

**Conclusion:**

Delayed-onset CMVR can occur in eyes treated with FAc implants, particularly in immunosuppressed patients. Recognition of atypical presentations, early PCR testing and timely antiviral therapy are essential to preserve vision.

## Introduction

Cytomegalovirus retinitis (CMVR) is a vision-threatening opportunistic infection most seen in individuals with significant systemic immunosuppression [1]. More recently, CMVR has also been recognised in eyes receiving local corticosteroid therapies, highlighting the complex relationship between ocular immune privilege, systemic immune status, and prolonged intraocular steroid exposure [2].

Fluocinolone acetonide (FAc) implants, specifically ILUVIEN (190 µg fluocinolone acetonide; Alimera Sciences Europe Ltd), deliver a continuous low-dose (0.2 µg/day) for up to three years [3]. They have become an important long-acting option for the management of uveitis with macular oedema and/ or retinal vasculitis, offering sustained inflammatory control with minimal systemic exposure [4]. While infectious events have been described following other intraocular steroid devices, most notably dexamethasone implant (OZURDEX; AbbVie Ltd., Berkshire, UK), the clinical behaviour of CMVR in the setting of long-acting FAc implants is not well defined, and FAc-associated CMVR has not been previously reported in the scientific literature [4, 5].

As the use of sustained-release corticosteroid implants expands, particularly as combined adjunctive treatment among patients who may also require systemic immunomodulatory therapy, recognising the range of potential retinal presentations in such eyes becomes increasingly relevant. We describe three patients who developed CMVR months after receiving FAc, each with different clinical patterns and management strategies. These cases highlight that long-acting intraocular corticosteroid exposure can lead to such presentations, emphasizing the need for careful examination and early diagnostic evaluation to avoid irreversible vision loss.

## Methods

This was a retrospective observational case series of three patients diagnosed with CMV retinitis following intravitreal FAc implantation between 2022 and 2024. During this period, approximately 160 FAc implants were administered at our centre. Patients were treated at the Uveitis department at Moorfields Eye Hospital, London, UK. A detailed history was obtained in each case, including systemic medications, immunosuppressive therapy and overall immune status. Demographic data, including age, sex, and time of diagnosis, were recorded. Clinical presentation, ocular findings, and final outcomes were studied.

## Results

### Case 1

**Fig. 1:**
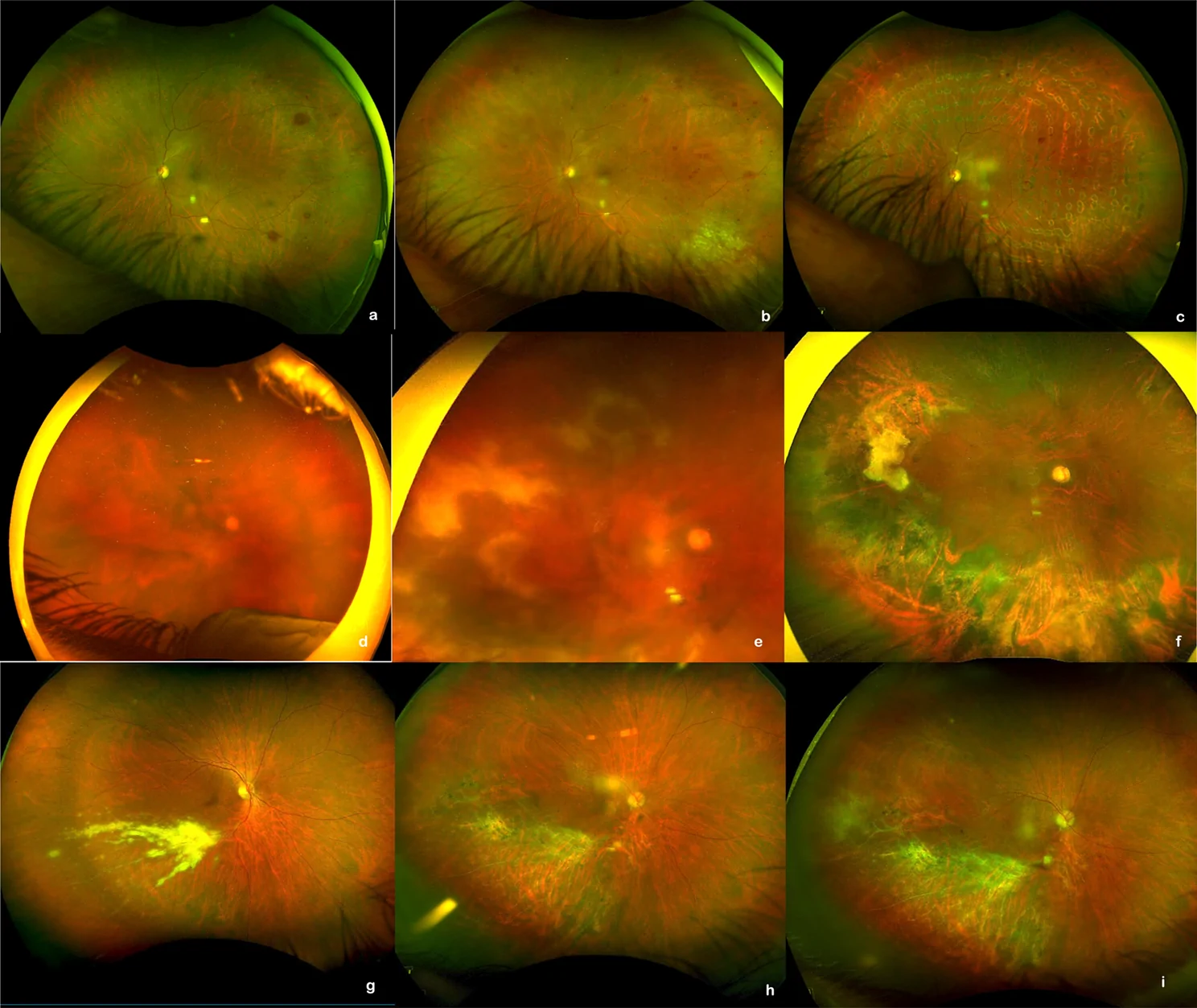
Montage of widefield pseudo-colour fundus photographs from three cases demonstrating delayed-onset cytomegalovirus (CMV) retinitis following fluocinolone acetonide (FAc) implantation. **(a)** Case 1: Baseline widefield fundus photograph of the left eye following FAc implantation, showing a few intraretinal haemorrhages in the superotemporal and inferotemporal quadrants, including two large blot haemorrhages. **(b)** Development of scattered intraretinal haemorrhages with resolution of the initial blot haemorrhages, along with a granular variant of CMV retinitis in the inferotemporal quadrant, associated with adjacent mid-peripheral retinal vasculitis. **(c)** Post-treatment image of the left eye showing panretinal photocoagulation scars following angiographic evidence of extensive capillary non-perfusion, with resolution of inferotemporal retinitis. **(d)** Case 2: Baseline widefield fundus photograph following FAc implantation, with hazy media due to vitritis. **(e)** Onset of fulminant necrotising CMV retinitis in the superotemporal quadrant, with increased vitritis contributing to further media haze. **(f)** Resolution of retinitis with extensive chorioretinal scarring and focal dystrophic calcification in the superotemporal quadrant at the previous site of CMV retinitis. (**g**) Case 3: Widefield fundus photograph demonstrating necrotising CMV retinitis in the inferotemporal quadrant in a patient with systemic lymphoma. **(h)** Fundus photograph after two years of quiescence, showing resolved CMV retinitis with chorioretinal scarring and the presence of an FAc implant placed for recurrent post-operative cystoid macular oedema. **(i)** Reactivation of CMV retinitis at the edge of a previous chorioretinal scar in the temporal quadrant, after FAc implantation

**Fig. 2:**
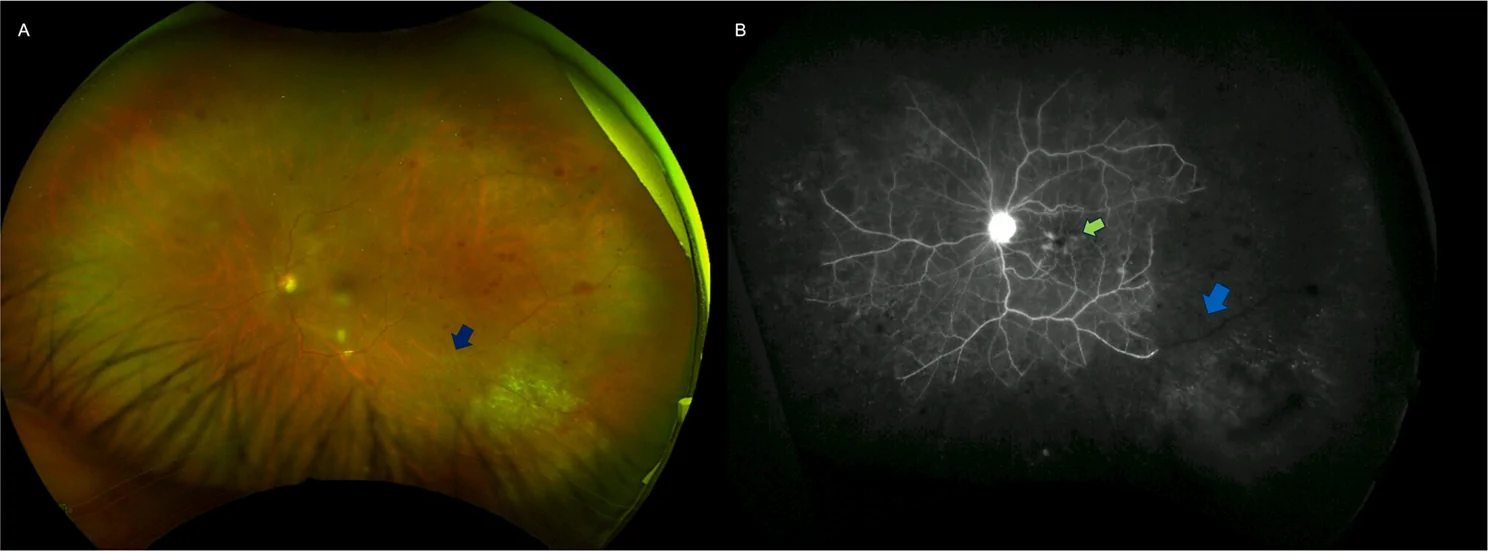
Ultra-widefield fluorescein angiogram of Case 1. (**A**) Widefield pseudo-colour fundus photograph of the left eye demonstrating multiple intraretinal haemorrhages, inferotemporal retinal vasculitis (blue arrow), and an adjacent area of granular CMV retinitis. (**B**) Widefield fundus fluorescein angiogram showing optic disc leakage, macular leakage (green arrow), occlusion of an inferotemporal vessel (blue arrow), and extensive 360° capillary non-perfusion

A 68-year-old man with biopsy-proven pulmonary sarcoidosis and bilateral intermediate uveitis, previously managed with vitrectomy and epiretinal membrane peel, multiple dexamethasone implants and a prior unilateral FAc for recurrent cystoid macular oedema, underwent bilateral intravitreal FAc (Fig. 1a). Approximately one year and two months later, he developed new onset of left-eye granular retinitis (Fig. 1b) with scattered intraretinal haemorrhages and severe occlusive retinal vasculitis on wide-field fluorescein angiography, showing extensive capillary non-perfusion (Fig. 2). The visual acuity was 20/200 in the left eye. Anterior-chamber PCR confirmed CMV. Oral valganciclovir (900 mg twice daily) was initiated but discontinued early due to worsening renal function, necessitating a switch to weekly intravitreal foscarnet. Because retinitis persisted and systemic antiviral therapy could not be continued, the left FAc implant was removed, combined with vitrectomy and panretinal photocoagulation for widespread ischaemia. The removal of the implant aimed at eliminating the effect of the long-acting steroid which was likely to perpetuate viral replication. The retinitis gradually resolved following implant removal and intravitreal antiviral therapy was ceased. Recurrent macular oedema was managed with difluprednate (0.05%) eyedrops. The eye remained structurally stable with resolved retinitis and laser scars, achieving a final visual acuity of 20/125 (Fig. 1c) (Table 1).

**Table 1:** Summary of treatment duration and CMVR diagnosis

Case	Age / Sex	Underlying Condition	Prior Interventions (Systemic and Local)	Time from FAc implantation to CMVR diagnosis	Visual acuity (presentation → final)
1	68 / M	Sarcoidosis (biopsy-proven)	Local immunosuppression; multiple Ozurdex; prior FAc implant	14 months	20/200 → 20/125
2	35 / M	Presumed ocular sarcoidosis	Adalimumab, MMF, tacrolimus, steroids; multiple Ozurdex	8 months	20/40 → 20/60
3	71 / M	Follicular lymphoma (post BMT)	Systemic immunosuppression; Ozurdex and FAc implant	8 months	20/40 → 20/80

### Case 2

A 35-year-old man with bilateral panuveitis of presumed ocular sarcoidosis aetiology on multiple systemic immunosuppressive agents, including adalimumab, mycophenolate, tacrolimus and oral prednisolone, underwent additional local corticosteroid therapy with multiple bilateral intravitreal dexamethasone implants, followed by bilateral intravitreal FAc (Fig. 1d). Approximately eight months after the right-eye FAc, he developed new right-eye CMV retinitis, presenting with a fulminant variant of necrotising retinitis and intraretinal haemorrhages (Fig. 1e). The best corrected visual acuity was 20/40 in right eye. Anterior-chamber PCR confirmed CMV. Oral valganciclovir 900 mg twice daily and intravitreal foscarnet were initiated, with complete resolution of the retinitis. The left eye remained uninvolved, and the right-eye CMV retinitis ultimately healed with dystrophic calcification and best corrected visual acuity of 20/60 (Fig. 1f) (Table 1).

### Case 3

A 71-year-old man with a history of follicular non-Hodgkins’s lymphoma treated with chemotherapy and autologous stem cell transplantation, developed bilateral CMV retinitis (Fig. 1g) with multiple recurrences, including ganciclovir-resistant disease. He required repeated intravitreal foscarnet, after which the left eye progressed to optic atrophy and chronic retinal detachment. The right eye CMV retinitis resolved following treatment. After two and half years, the right eye underwent cataract surgery, and recurrent macular oedema with mild vitreomacular traction was managed with dexamethasone implant followed by FAc implantation (Fig. 1h). Eight months after FAc, he experienced right eye reactivation of CMV retinitis, visual acuity being 20/40, confirmed by anterior-chamber PCR and treated with further intravitreal foscarnet, resulting in ocular stabilisation and final visual acuity of 20/80 (Fig. 1i) (Table 1).

## Discussion

CMVR typically occurs in the setting of systemic immunosuppression, but increasing evidence shows that local intraocular corticosteroid delivery can also create a permissive environment for CMV reactivation [1]. Most published cases describe CMVR developing within weeks of a dexamethasone implant, suggesting that short-term, high-intensity local steroid exposure may be sufficient to impair retinal immune surveillance [2–4]. FAc implants, such as ILUVIEN, differ substantially from the dexamethasone implant in terms of pharmacokinetics, as they release a much lower daily dose (0.2 µg/day release) that is sustained for up to three years, creating a prolonged period of localized immunosuppression [3, 5–7].

Corticosteroids suppress antigen presentation, inhibit CD4⁺ T-cell activity and down-regulate cytokine transcription. Prior work has shown that even brief steroid exposure may enable CMV replication in latently infected retinal cells [2]. Age-related immune decline, or immunosenescence, may further increase susceptibility, as reduced CD4⁺ T-cell proliferative capacity and rising CMV seroprevalence with age create an environment in which modest reductions in local immunity may allow viral reactivation [2].

Published cases of post-steroid CMVR have almost exclusively followed intravitreal triamcinolone acetonide and dexamethasone implant, with onset reported at approximately two to eight weeks after injection [2–4]. In contrast, CMVR associated with low-dose FAc implants has not been reported previously although there are two cases of CMVR following 0.59 mg fluocinolone acetonide implants [5]. The three patients in this series developed CMVR months after FAc placement, indicating that prolonged low-dose steroid exposure can also predispose to infection, particularly when combined with systemic immunomodulatory therapy. Two patients were receiving systemic immunosuppression, suggesting that the cumulative effect of systemic and sustained local steroid delivery may lower the threshold for viral reactivation, which is reinforced by the fact that only the eye receiving the implant experienced the viral reactivation.

The phenotype of retinitis varied across patients: one eye demonstrated a granular variant with occlusive vasculitis, another developed fulminant necrotising retinitis, and the third experienced reactivation at the margin of a previous CMV scar. Notably, CMVR remained unilateral in the first two cases despite bilateral FAc implants, raising the possibility that asymmetric viral latency, local tissue susceptibility, or previous retinal injury may determine the site of reactivation.

Management required tailored antiviral strategies, including intravitreal foscarnet, oral valganciclovir when tolerated, and, in one case, implant removal due to persistent viral activity and renal limitations on systemic therapy. Structural stabilisation was achieved in all eyes, although visual outcomes reflected the extent of retinal necrosis and pre-existing pathology.

This case series highlights an emerging and clinically relevant risk of delayed-onset CMVR following treatment with the FAc implant, particularly in patients with systemic immunosuppression or age-related immune vulnerability. Not all patients in our series were on systemic IMT, suggesting that local FAc-induced immunosuppression may, in some patients, be sufficient to permit viral reactivation. Ophthalmologists should maintain heightened vigilance for early symptoms or subtle fundus changes in such patients, even when the contralateral eye remains unaffected. Regular surveillance may allow prompt diagnosis and help prevent irreversible visual loss.

## Conclusion

CMV retinitis may manifest as a delayed-onset infection in eyes following treatment with an FAc implant, particularly in patients receiving systemic immunosuppression and following local intravitreal steroids because of cumulative local immunosuppressive effect. Awareness of this possibility is important when evaluating new retinal whitening or occlusive vasculitis changes, even months after implantation. Patients should be counselled regarding the potential risk, the chronic and recurrent nature of CMV disease, and the possible need for antiviral therapy, implant removal, or additional interventions to preserve vision. 

## Data Availability

No datasets were generated or analysed during the current study.
